# Trajectories of positive, negative and general psychopathology symptoms in first episode psychosis and their relationship with functioning over a 2-year follow-up period

**DOI:** 10.1371/journal.pone.0187141

**Published:** 2017-11-02

**Authors:** Edimansyah Abdin, Siow Ann Chong, Janhavi Ajit Vaingankar, Chao Xu Peh, Lye Yin Poon, Sujatha Rao, Swapna Verma, Mythily Subramaniam

**Affiliations:** 1 Research Division, Institute of Mental Health, Singapore; 2 Department of Early Psychosis Intervention, Institute of Mental Health, Singapore; Chiba Daigaku, JAPAN

## Abstract

**Background:**

Few studies have examined the trajectories of symptom severity in first episode psychosis (FEP) and their impact on functioning. This study aimed to identify discrete trajectories of positive, negative and general psychopathological symptoms and functioning, determine predictors of the identified symptom trajectories and subsequently investigate the relationship between symptom and functioning trajectories over the 2-year follow-up period.

**Methods:**

Data were extracted from the Singapore Early Psychosis Intervention Programme clinical database. Trajectories of the Positive and Negative Syndrome Scale and Global Assessment of Functioning (GAF) scale over the two-year follow up were modelled using latent class growth curve modelling.

**Results:**

Two distinct trajectories (*early response and stable trajectory* and *delayed response trajectory*) for positive symptoms, four distinct trajectories (e*arly response and stable trajectory*, *early response and relapse trajectory*, *slower response and no response trajectory* and *delayed response trajectory*) for negative and general psychopathology symptoms and three distinct trajectories for functioning (*high functioning trajectory*, *moderately stable functioning trajectory* and *deterioration in functioning trajectory*) were identified in our sample. Compared to individuals in the *early response and stable trajectory*, those in the *delayed response trajectory* for positive and negative symptoms, *early response and relapse* for negative and general psychopathology symptoms and *slower response and no response* trajectories for general psychopathology symptoms were significantly associated with higher odds of having *deterioration in functioning* over time. Poor symptom trajectories were also significantly predicted by younger age, male gender, unemployed and economically inactive status, lower education, longer duration of untreated psychosis and diagnosis of schizophrenia spectrum and delusional disorders.

**Conclusions:**

The results confirm that the symptoms trajectories among patients with FEP are heterogeneous and suggest that a small group of patients may be at higher risk of deterioration in symptom severity and functioning over the 2-year follow-up.

## Introduction

Schizophrenia is one of the leading causes of long-term disability [1], and affects about 25 million people worldwide [2]. Schizophrenia and other psychoses are highly disabling, recurrent, and most often lifelong conditions with substantial costs to the patient and their family members especially if they delay or fail to seek treatment. Various early psychosis intervention studies have reported that early treatment leads to better recovery [3–8]. Observation of different trajectories of symptoms during treatment has been recognized as a key focus within schizophrenia research that provides valuable information for improving treatment outcome [9–13]. This approach aims to capture inherent patterns of response to treatment longitudinally by characterizing the subgroups of patients with similar course of response and examining illness characteristics and their predictors [13] using growth curve modelling techniques such as latent class growth analysis [14, 15]. The advantage of this approach is that if a subgroup with a different trajectory is identified which differs in terms of predictors and treatment outcomes, it may create an opportunity for new treatment strategies specific to that subgroup to improve recovery [9, 16].

Evidence from longitudinal studies suggests that trajectories of the course of symptom severity among individuals with first episode psychosis (FEP) are heterogeneous [9–12, 17–19]. Early work by Levine & Rabinowitz [10] suggested five distinct trajectories of treated symptom severity of positive and negative symptoms (PANSS) total scores in a clinical trial of haloperidol and risperidone among a first-episode psychosis sample. It comprised three groups with various baseline PANSS scores that did not surpass 30% PANSS improvement over time, a fourth group with 43.5% PANSS improvement that remained stable and a fifth group that showed marked improvement with more than 50% PANSS improvement over time. Few other studies have used a similar approach to look at the distinct trajectories of symptoms of patients with FEP. For instance, Case et al. [13] identified four trajectories over a 12-week follow-up period using data from a randomized, double-blind study of patients with schizophrenia or schizoaffective disorders treated with risperidone or olanzapine. In a naturalistic study from an early psychosis intervention programme, Schennach et al. [12] found five distinct trajectories of antipsychotic response; Austin et al [9] identified five distinct trajectories for positive symptoms namely *response*, *delayed response*, *relapse*, *non-response* and *episodic response* and four distinct trajectories for negative symptoms which were *response*, *delayed response*, *relapse and non-response*, while Gee et al. [16] identified four distinct trajectories for negative symptoms—*minimal decreasing*, *mild stable*, *high decreasing* and *high stable*. Previous studies conducted in Western populations have shown that socio-demographic factors such as younger age and male gender and clinical factors such as having schizophrenia diagnosis, poor functioning at baseline, and a longer duration of untreated psychosis (DUP) are associated with poor symptom trajectories [9, 20]. Few studies have been conducted in Asian populations to identify the nature of symptom trajectories and its impact on functioning among patients with FEP. Given that identification of different categories is based on temporal patterns of symptom change and its associated factors could impact illness course [9, 16], it is important to replicate this study in an Asian population. Hence, the aims of the current study were to identify discrete trajectories of positive, negative and general psychopathological symptoms collected over a 2 years follow-up in the Singapore Early Psychosis Intervention Programme (EPIP); determine predictors of the identified symptom trajectories and subsequently investigate the relationship between symptom trajectory and functioning over the 2 year follow-up period.

## Methods

Singapore is an island state in Southeast Asia with a population of 5.6 million. The majority of its population is Chinese (74.3%), followed by the Malays (13.4%), Indians (9.1%) and other ethnic groups (3.3%)[21]. The Singapore Early Psychosis Intervention Programme (EPIP) is a nationwide programme that was launched in 2001 at the Institute of Mental Health, the only state psychiatric hospital in Singapore. Patients included in this programme met the following criteria: (1) age between 15 and 40 years, (2) first episode psychotic disorder with no prior or minimal treatment i.e., less than 12 weeks of antipsychotic medications, (3) no current history of substance abuse, and (4) no history of major medical or neurological illness. This programme which has both an early detection as well as an early intervention component provides phase-specific multidisciplinary care [22]. The multidisciplinary approach comprises a comprehensive, integrated, patient-centred program that provides intensive care to patients in the early stages of the illness to improve outcomes and change the trajectory of the disease. Case management is an integral aspect of EPIP’s care model and each patient has a case manager who develops a comprehensive phase-specific care plan in collaboration with each patient. The case manager provides supportive counselling, psychoeducation, as well as coordinates the various healthcare services to ensure the continuity of care through the different phases of the illness. If required, patients are also referred to the psychologist or occupational therapist for various psychosocial interventions. Psychopharmacological treatment is based on a treatment algorithm that emphasizes the use of antipsychotic monotherapy at low dose [3, 22]. Patients who were accepted into EPIP from 2001 to 2012 were included in current study. The study was approved by the institutional ethics review boards of participating institutions which include the National Healthcare Group, Domain Specific Review Board. Consent from patients or parents (for patients aged below 21 years) was not required in the current study because it was based on an anonymized clinical database maintained by the hospital. However, permission to use the dataset for the study was approved by the institutional ethics review board i.e. the National Healthcare Group, Domain Specific Review Board (DSRB) (DSRB Reference: 2016/00017).

**Assessments:** Diagnosis was established using the Structured Clinical Interview for Diagnostic and Statistical Manual of Mental Disorders, 4^th^ Edition (DSM-IV) Axis I disorders at the first contact (baseline) with EPIP. Information relating to DUP, age, gender, marital status, ethnicity and education status was obtained from patients and relatives by the clinicians and case managers using a semi-structured questionnaire. DUP was defined as the time in months between onset of psychotic symptoms (i.e. delusions, hallucinations, disorganized behaviour) and the time when a definitive diagnosis and treatment were established. As part of their routine assessment, severity of psychopathology symptoms and level of functioning at baseline and 3, 6, 12 and 24 months were assessed using PANSS [23] and Global Assessment of Functioning Scale (GAF)[24], respectively. These ratings were conducted by experienced psychiatrists who were trained in the use of the rating instruments. The inter-rater reliability for PANSS in our sample was 0.94.

### Statistical analyses

All statistical analyses were performed using SAS version 9.2 (SAS Institute, Cary, NC) and MPLUS software version 7.11. Mean and standard deviations were calculated for continuous variables, and frequencies and percentages were calculated for categorical variables. A latent class growth analysis (LCGA) was used to identify distinct trajectories for positive, negative, general psychopathology symptom severity and functioning over the 2-year follow-up period. LCGA is a statistical technique developed by Nagin [25] for identifying distinct homogeneous subpopulations with similar trajectories of growth over time (known as latent classes) within longitudinal data collected from a larger heterogeneous population [15]. Trajectory is defined as a group of people who have a homogenous symptom profile within the group and significantly dissimilar (i.e. heterogeneous) from other groups [9, 11]. Missing data were handled using full information maximum likelihood under the assumption that data were missing at random. The best-fitting model was chosen according to Bayesian Information Criterion (BIC), Akaike Information Criterion (AIC), Lo-Mendell-Rubin Likelihood Ratio Test (LMR-LRT), and Bootstrapped Likelihood Ratio Test (BLRT). Lower BIC and AIC values suggest more parsimonious model fit while a significant LMR-LRT or BLRT value suggests that additional a K class model fits the data better than a K-1 model, where K refers to the number of classes. Interpretability of the successive models was also considered alongside fit indices. Multinomial logistic regression analysis was used to explore significant factors associated with latent class trajectory identified in LCGA model. Age, gender, education level, employment status, marital status, ethnicity, DUP, and diagnosis were included as predictors in the regression models. The effects of latent class symptoms trajectory, baseline socio-demographic, clinical factors on functioning over time were also estimated using multinomial logistic regression analysis. Statistical significance was set at p value < 0.05.

## Results

### Characteristics of sample

Sociodemographic and clinical characteristics of the patients are presented in Table 1. The sample comprised 875 males (50.8%) and 849 females (49.3%) with a mean (SD) age of 28 (6.6) years and a range of 14 to 40 years. One thousand and two hundred eighty three patients (74.4%) were diagnosed with schizophrenia spectrum and delusional disorders, 8.8% (n = 151) with affective psychosis and 16.8% (n = 290) with brief psychotic disorder and psychotic disorder Not Otherwise Specified (NOS). The mean DUP (SD) was 14.7 (26.4) months and median was 5 months. Majority of patients (77.8%) were never married. 65.8%, 64.7%, 63% and 56.7% of the sample (n = 1724) completed the 3-month, 6-month, 12-month and 24-month follow-up assessment, respectively. As compared to those who had completed follow-up assessment on PANSS scores at 2 years follow-up, those who had missing data were more likely to be males, of Malay ethnicity (vs. Chinese), and less likely to be married (vs. single) and had a diagnosis of brief psychotic disorder and psychotic disorder NOS (vs. schizophrenia spectrum and delusional disorder).

**Table 1:** Sociodemographic profiles.

	n	%	Mean	SD
Age			27.7	6.6
Sex				
Female	849	49.3		
Male	875	50.8		
Race				
Chinese	1303	75.6		
Malay	253	14.7		
Indian	125	7.3		
Others	43	2.5		
Marital status				
Single/unmarried	1341	77.8		
Married	316	18.3		
Separated/Divorced	67	3.9		
Employment status				
Employed	479	27.8		
Unemployed	768	44.6		
Economically inactive*	477	27.7		
Education				
Primary and below	244	14.2		
Secondary	612	35.5		
Tertiary	868	50.4		
SCID				
Schizophrenia spectrum and delusional disorder	1283	74.4		
Affective psychosis	151	8.8		
Brief Psychotic Disorder and Psychotic disorder NOS	290	16.8		
DUP since onset of symptoms			14.7	26.3
Baseline PANSS Positive			19.6	6.0
Baseline PANSS Negative			12.8	7.0
Baseline PANSS GPS			33.6	9.7
Baseline GAF disability			45.6	13.4
Baseline GAF symptoms			40.8	14.3

*Economically inactive = Student and Homemaker

### Trajectories of positive symptoms

**Fig 1:**
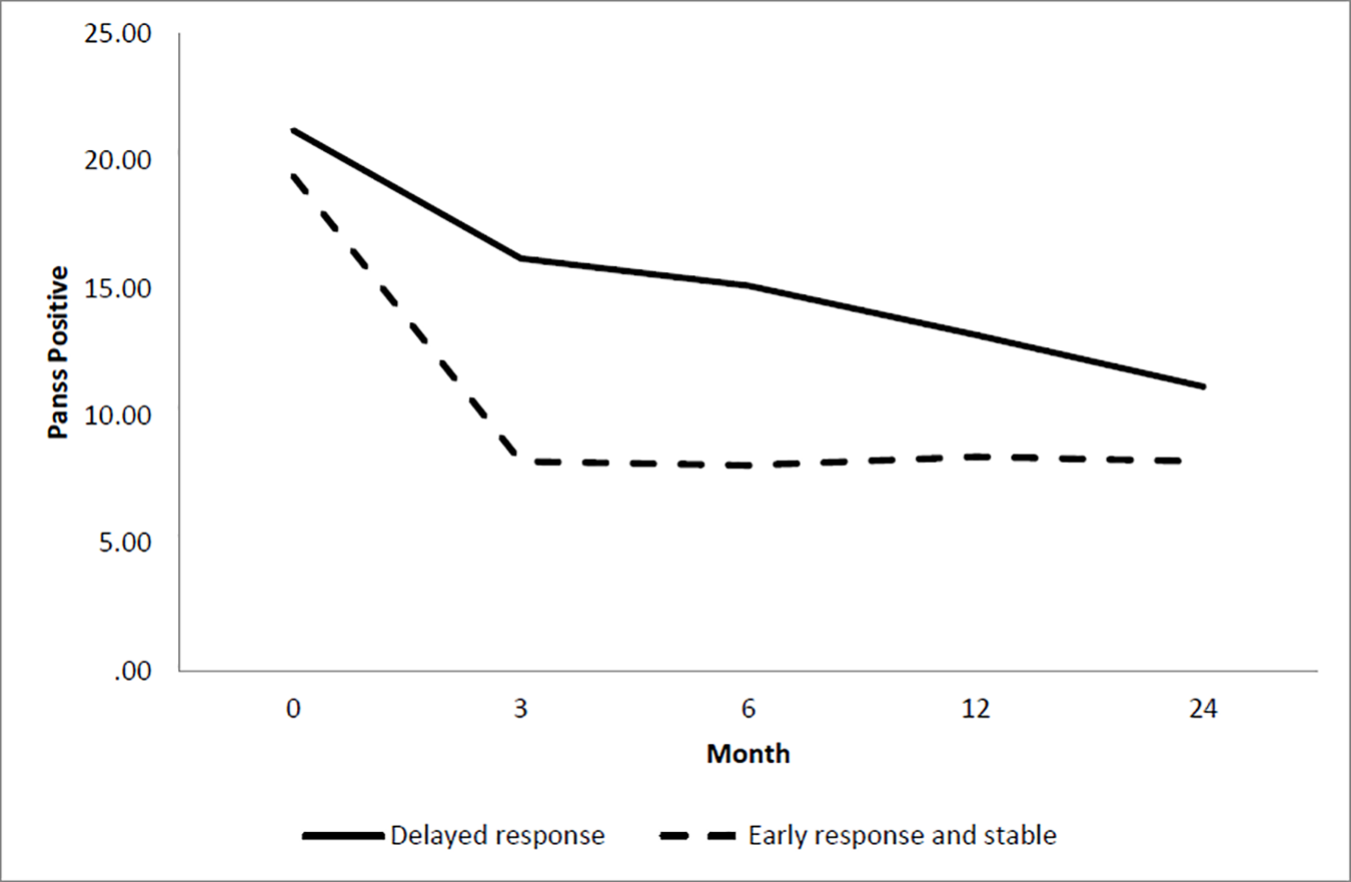
Latent class trajectory of PANSS positive symptoms.

Table 2 shows the goodness-of-fit statistics of different classes of PANSS positive, negative, general psychopathology symptoms and GAF functioning. Our results revealed that a two-class solution provided lower BIC and AIC values than the one class and was supported with a significantly better fit for positive symptoms according to both the LMR-LRT (136.2, p value = 0.0014) and BLRT (838.5, p value <0.001) statistics. These two classes consist of *early response and stable trajectory* (Class Sizes (CS) = 87.7%) which was characterized by a significant reduction in positive symptoms within the first 3-months followed by maintenance of a low level of symptom severity over the remaining months as well as a *delayed response trajectory* (CS = 12.3%) which was characterized by an initial response within 3-months followed by steady reduction in symptoms over the 2 years follow-up (Fig 1). Compared to individuals in the *early response and stable trajectory*, those in *delayed response trajectory* were more likely to be male (OR = 1.5), having secondary education (OR = 1.1), longer DUP (OR = 1.01) and less likely to be diagnosed with affective psychosis (OR = 0.3) and brief psychotic disorder and psychotic disorder NOS (OR = 0.6) (Table 3).

**Table 2:** Goodness-of-fit statistics of different classes of PANSS Positive, Negative, General Psychopathology symptoms and GAF functioning.

Number of classes	Number of free parameters	Log likelihood	AIC	BIC	Entropy (%)	LMR-LRT statistic	p value	BLRT statistic	p value
Positive symptoms									
1	7	-18105	36224	36262.2	-				
2	10	-17685.7	35391.5	35446.0	78.6	136.2	0.001	838.5	<0.001
3	13	-17460.2	34946.4	35017.3	79.1	451.1	0.637	451.1	<0.001
4	16	-17293.8	34619.7	34706.9	79.6	332.7	0.087	332.7	<0.002
5	19	-17169.5	34377.1	34480.7	79.3	248.6	0.277	248.6	<0.001
Negative symptoms									
1	7	-18521.5	37057.0	37095.2					
2	10	-17638.4	35296.9	35351.4	88.6	1766.2	0.001	1766.2	<0.001
3	13	-17354.5	34735.0	34805.9	90.8	567.8	0.0002	567.8	<0.001
4	16	-17135	34302	34389.2	89.2	439.0	0.030	439.0	<0.001
5	19	-17032.061	34102.12	34205.7	86.9	351.1	0.499	205.9	<0.001
General Psychopathology symptoms									
1	7	-21107.3	42228.6	42266.7					
2	10	-20696.8	41413.6	41468.1	0.814	821	0.0001	821	<0.001
3	13	-20567.4	41160.9	41231.8	0.802	258.7	0.026	258.7	<0.001
4	16	-20471.5	40975.0	41062.3	0.764	191.9	0.036	191.9	<0.001
5	19	-20414.3	40866.6	40970.2	0.751	114.5	0.216	114.5	<0.001
GAF functioning									
1	7	-23745.2	47504.4	47542.6					
2	10	-23546.1	47112.3	47166.8	0.508	398.1	<0.001	381.1	<0.001
3	13	-23506.6	47039.1	47110.0	0.601	79.1	0.014	75.755	0.016
4	16	-23483.6	46999.2	47086.4	0.565	45.9	0.136	44.0	0.145
5	19	-23467.6	46973.2	47076.8	0.503	31.9	0.108	30.6	0.109

**Table 3:** Predictors of positive, negative and general psychopathology symptoms trajectories.

	Positive symptoms		Negative symptoms						General psychopathology symptoms					
	Delayed response		Early response and relapse		Slower response and no response		Delayed response		Early response and relapse		Delayed response		Slower response and no response	
	OR	(95% CI)	OR	(95% CI)	OR	(95% CI)	OR	(95% CI)	OR	95% CI	OR	95% CI	OR	95% CI
Age	0.9	(0.9–1.0)	0.9	(0.9–1.0)	0.95	(0.8–0.9)*	0.95	(0.9–0.98)*	0.9	(0.9–1.0)	0.9	(0.9–1)	0.9	(0.9–1.0)
Male vs. Female	1.5	(1.1–2)*	1.4	(0.9–2.2)	1.7	(0.6–4.8)	1.5	(1.1–2.2)*	1.1	(0.7–1.6)	2.5	(1.2–5.4)*	1.4	(1.0–2.1)*
Married vs. single	0.8	(0.5–1.3)	0.6	(0.3–1.3)	.	.	0.8	(0.4–1.4)	0.9	(0.5–1.7)	0.5	(0.1–2.6)	1.0	(0.6–1.8)
Separated vs. single	1.1	(0.5–2.5)	0.2	(0–1.6)	.	.	0.3	(0.1–1.4)	0.5	(0.1–2.1)	1.8	(0.4–8.9)	0.6	(0.2–1.9)
Unemployed vs. Employed	1.1	(0.7–1.6)	1.8	(1–3.2)*	7.7	(0.9–61.8)	2.3	(1.4–3.7)*	1.6	(0.9–2.7)	4.3	(1.2–14.8)*	2.8	(1.7–4.8)*
Economically inactive vs. Employed	1.3	(0.8–2)	1.7	(0.9–3.2)	1.9	(0.2–18.8)	1.3	(0.8–2.3)	1.5	(0.8–2.7)	4.9	(1.3–18)*	2.5	(1.4–4.4)*
Malay vs. Chinese	0.8	(0.5–1.2)	1.1	(0.6–2)	1.8	(0.6–5.5)	0.9	(0.5–1.4)	1.1	(0.6–2.0)	1.2	(0.5–2.8)	0.6	(0.3–1.1)
Indian vs. Chinese	1.0	(0.6–1.8)	1.7	(0.8–3.4)	0.7	(0.1–5.7)	0.7	(0.3–1.5)	1.4	(0.7–2.9)	1.3	(0.4–4.3)	1.4	(0.8–2.6)
Others vs. Chinese	1.5	(0.6–3.5)	2.3	(0.9–6.4)	.	.	0.5	(0.1–2.3)	2.4	(0.9–6.5)	.	.	2.1	(0.9–5.1)
Primary vs. Tertiary	1.1	(0.7–1.8)	1.9	(1.1–3.5)*	8.5	(2–36.2)*	1.6	(0.9–2.7)	1.1	(0.6–2.0)	2.0	(0.8–4.9)	1.4	(0.8–2.3)
Secondary vs. Tertiary	1.5	(1.1–2)*	1.4	(0.9–2.2)	4.1	(1.1–15.7)*	1.7	(1.2–2.5)*	1.1	(0.7–1.7)	1.1	(0.5–2.3)	1.2	(0.9–1.8)
DUP	1.01	(1.0–1.01)*	1.0	(0.9–1.0)	1.01	(1.0–1.03)*	1.01	(1.0–1.01)*	1.01	(1.0–1.01)*	1.0	(0.9–1.0)	1.0	(0.9–1.0)
Diagnoses														
Affective psychosis vs. Schizophrenia spectrum & delusional disorder	0.3	(0.2–0.7)*	0.2	(0.1–0.8)*	.	.	0.5	(0.3–1.1)	0.5	(0.2–1.2)	1.0	(0.3–3.1)	0.9	(0.5–1.8)
Brief psychotic disorder & psychotic disorder NOS vs. Schizophrenia spectrum & delusional disorder	0.6	(0.4–0.9)*	0.2	(0.1–0.6)*	.	.	0.5	(0.3–0.9)*	0.4	(0.2–0.8)*	0.7	(0.2–1.9)	0.4	(0.2–0.8)*

Note: Early response and stable is reference group.

* = Significant p value < 0.05.

### Trajectories of negative symptoms

**Fig 2:**
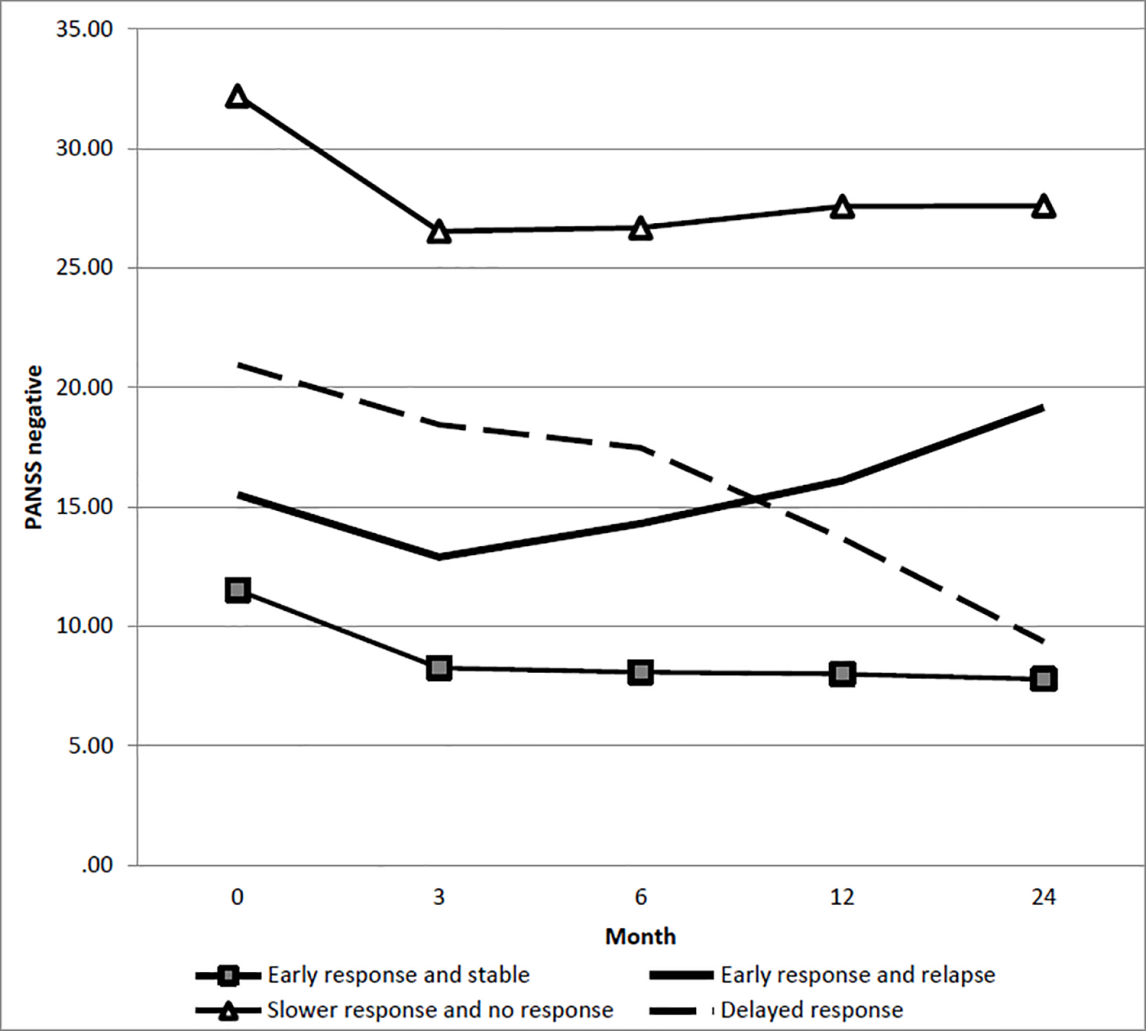
Latent class trajectory of PANSS negative symptoms.

A four-class solution was identified as optimal fit for negative symptoms (Table 2). The first trajectory (*early response and stable trajectory*; CS = 84%) was characterized by a significantly low level of negative symptoms in the early stage, initial decrease and stabilization over the remaining months. The second trajectory (*early response and relapse trajectory*; CS = 5.9%) was characterized by initial decrease of the symptoms within the 3-month and then relapse over the remaining months. The third trajectory (*slower response and no response* trajectory; CS = 8.9%) was characterized by early response with reduction in the negative symptoms within the 3-month period followed by no response to treatment in the remaining months. The fourth trajectory (*delayed response trajectory*; CS = 1.2%) was characterized by initial response within 3-months, which was maintained between 3 and 6 months, followed by reduction throughout the remaining months (Fig 2). Compared to individuals in the *early response and stable trajectory*, those in *early response and relapse trajectory* were more likely to be unemployed (OR = 1.8), having primary education (OR = 1.9) and less likely to be diagnosed with affective psychosis (OR = 0.2), brief psychotic disorder and psychotic disorder NOS (OR = 0.2). Those in the *slower response and no response trajectory* were more likely to have primary (OR = 8.5) and secondary education (OR = 4.1), longer DUP (OR = 1.01) and were less likely to be of younger age (OR = 0.9). Those in the *delayed response trajectory* were more likely to be male (OR = 1.5), unemployed (OR = 2.3), having secondary education (OR = 1.7), longer DUP (1.01), and less likely to be of younger age (OR = 0.95) and diagnosed with brief psychotic disorder and psychotic disorder NOS (OR = 0.5) (Table 3).

### Trajectories of general psychopathology symptoms

**Fig 3:**
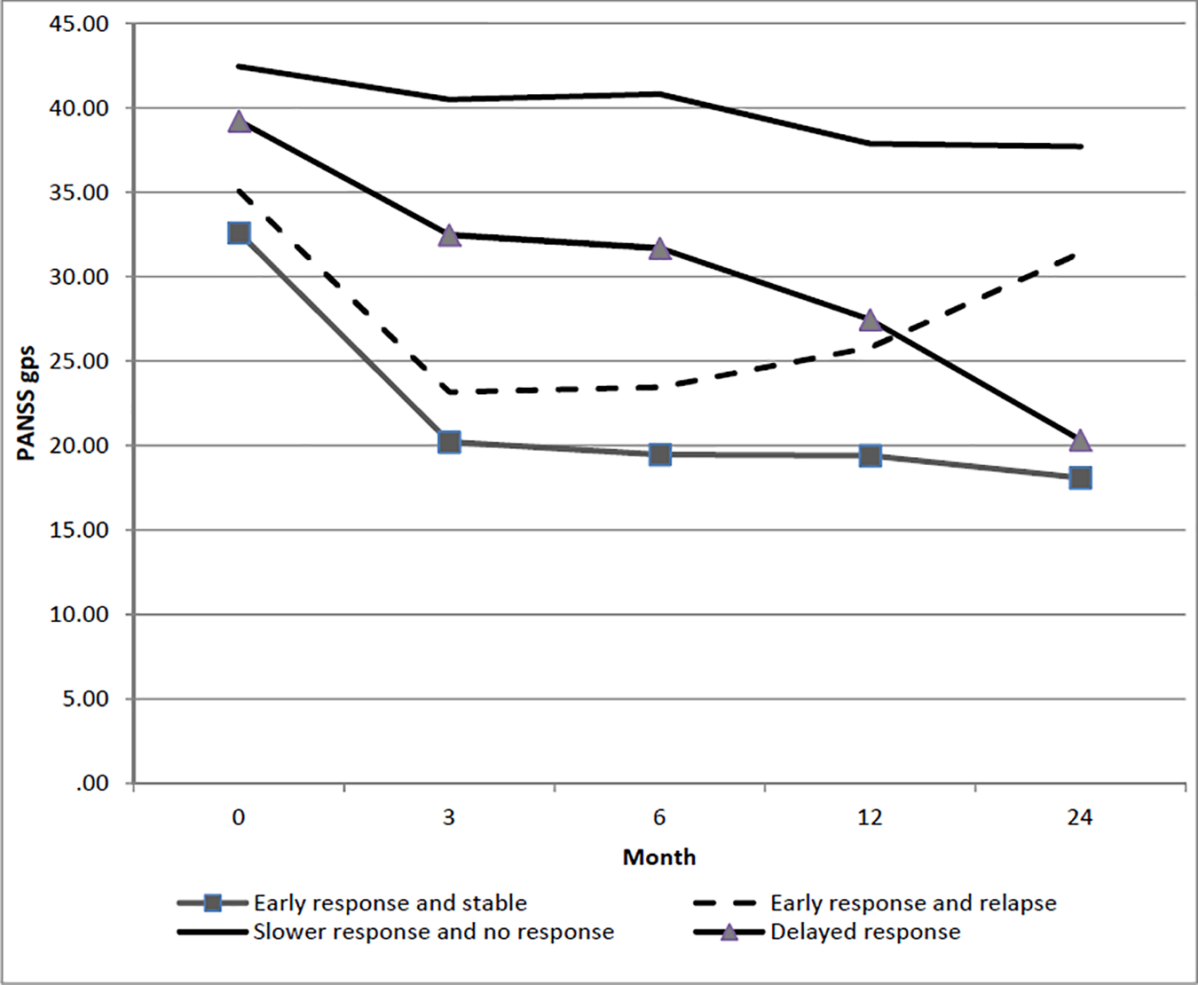
Latent class trajectory of general psychopathological symptoms.

Four-class solution was also observed for general psychopathology symptoms (Table 2). The first and second trajectories were labelled as *early response and stable trajectory* (CS = 82.1%) and *early response and relapse trajectory* (CS = 6.4%). The third trajectory (*delayed response trajectory*; CS = 2.2%) was characterized by initial response trajectory within 3 months, which was maintained between 3 and 6 months, followed by reduction throughout the remaining months. The fourth trajectory was labelled as *slower response and no response trajectory* (CS = 9.2%) due to minimal reduction of the symptoms with significantly higher symptoms throughout the entire follow-up period (Fig 3). Compared to individuals in the *early response and stable trajectory*, those in the *early response and relapse trajectory* were more likely to have a longer DUP (OR = 1.01) and less likely to be diagnosed with brief psychotic disorder and psychotic disorder NOS (OR = 0.4). Those in the *delayed response trajectory* were more likely to be male (OR = 2.5), unemployed (OR = 4.3) or economically inactive (OR = 4.9) whilst those in *slower response and no response trajectory* were more likely to be male (OR = 1.4), unemployed (OR = 2.8), economically inactive (OR = 2.5) and less likely to be diagnosed with brief psychotic disorder and psychotic disorder NOS (OR = 0.4) (Table 3).

### Impact of symptom trajectories on functioning

**Fig 4:**
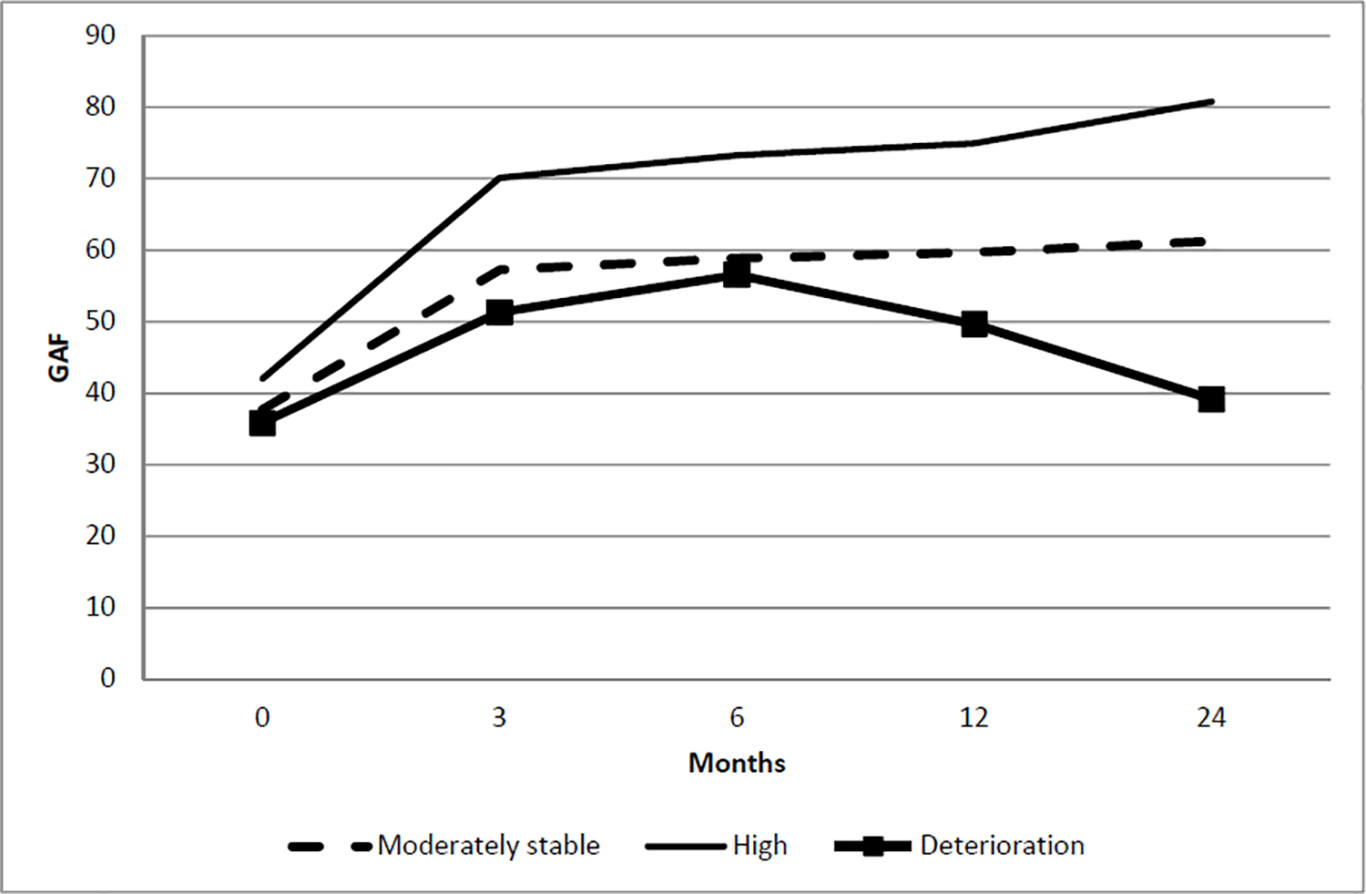
Latent class trajectory of functioning.

Three distinct trajectories were identified for functioning: (a) *high functioning trajectory* (CS = 63.9%)–a significant improvement in functioning over time, (b) *moderately stable functioning trajectory* (CS = 33%)—initial increase within 3 months to 6 months, which remained moderately stable throughout the remaining months and (c) *deterioration in functioning* (CS = 3.1%)—minimal improvement in functioning within 6 months and significant drop in functioning throughout the remaining months (Fig 4). Compared to individuals in the *early response and stable trajectory*, those belonging to the *delayed response trajectory* for positive and negative symptoms, *early response and relapse* for negative and general psychopathology symptoms and *slower response and no response trajectory* for general psychopathology symptoms were more likely to have *deterioration in functioning trajectory*. *Delayed response trajectory* in positive symptoms, *early response and relapse trajectory* for negative and general psychopathology symptoms as well as *slower response and no response trajectory* in general psychopathology symptoms were more likely to have *moderately stable functioning trajectory*. Compared to individuals in *early response and stable trajectory*, we also found that those belonging to *moderately and stable functioning trajectory* were more likely to be unemployed with longer DUP and less likely to be diagnosed with brief psychotic disorder and psychotic disorder NOS (Table 4).

**Table 4:** Relationship between latent class trajectory, sociodemographic, clinical factors and functioning overtime.

	Moderately stable vs High			Deterioration vs High		
	OR	95% CI	p value	OR	95% CI	p value
Positive symptoms						
*Early response and stable*	Ref			Ref		
*Delayed response*	2.80	(1.9,4.12)	<0.001	3.46	(1.53,7.84)	0.003
Negative symptoms						
*Early response and stable*	Ref			Ref		
*Early response and relapse*	4.57	(2.54,8.22)	<0.001	3.41	(1.21,9.59)	0.020
*Delayed response*	2.42	(0.42,14.08)	0.324	9.18	(1.07,79.08)	0.044
*Slower response and no response*	1.00	(0.66,1.54)	0.987	0.84	(0.27,2.59)	0.762
General psychopathology symptoms						
*Early response and stable*	Ref			Ref		
*Early response and relapse*	22.02	(9.88,49.07)	<0.001	227.21	(76.32,676.42)	<0.001
Delayed response*	.			.		
*Slower response and no response*	2.73	(1.78,4.17)	<0.001	5.87	(1.82,18.96)	0.003
Age	0.99	(0.97,1.01)	0.258	0.98	(0.92,1.05)	0.571
Gender						
Male vs Female	1.18	(0.93,1.51)	0.168	1.97	(0.96,4.07)	0.065
Marital status						
Married vs. single	0.84	(0.58,1.21)	0.352	1.57	(0.54,4.57)	0.412
Separated/divorced vs. single	1.12	(0.62,2.04)	0.707	0.94	(0.1,8.69)	0.960
Employment status						
Unemployed vs. Employed	1.36	(1.02,1.81)	0.038	1.63	(0.64,4.17)	0.305
Economically inactive vs. Employed	0.83	(0.58,1.17)	0.282	1.12	(0.39,3.23)	0.838
Ethnicity						
Malay vs. Chinese	0.92	(0.66,1.29)	0.638	1.63	(0.69,3.87)	0.268
Indian vs. Chinese	0.79	(0.49,1.28)	0.345	0.84	(0.25,2.9)	0.788
Others vs. Chinese	0.85	(0.39,1.83)	0.670	1.26	(0.21,7.4)	0.799
Education						
Primary vs. Tertiary	1.14	(0.8,1.64)	0.465	1.23	(0.49,3.09)	0.665
Secondary vs. Tertiary	1.26	(0.97,1.63)	0.078	0.93	(0.44,1.97)	0.854
DUP symptoms	1.01	(1,1.01)	0.028	1.01	(1,1.02)	0.140
SCID Diagnoses						
Affective psychosis vs. Schizophrenia spectrum & delusional disorder	0.82	(0.53,1.27)	0.375	0.48	(0.1,2.36)	0.370
Brief psychotic disorder & psychotic disorder NOS vs. Schizophrenia spectrum & delusional disorder	0.69	(0.49,0.98)	0.037	0.77	(0.25,2.38)	0.651

Note: High functioning is reference group.

* = Parameter estimates were not estimated due to low number

## Discussion

This study used LCGA to stratify patients with FEP into distinct trajectories based on symptom severity and functioning. The results confirm those from previous studies [9–12, 17–19] that trajectories of treated symptom severity among patients with FEP are heterogeneous. Our data suggests that there is evidence of two distinct trajectories (*early response and stable trajectory* and *delayed response trajectory)* for positive symptoms and four distinct trajectories (*early response and stable trajectory*, *early response and relapse trajectory*, *slower response and no response trajectory* and *delayed response trajectory*) for negative and general psychopathology symptoms among patients with FEP. A previous study on the OPUS cohort by Austin et al [9] which examined patients with FEP over 10 years of follow-up using a similar statistical methodology had identified five distinct trajectories (*response*, *delayed response*, *relapse*, *no response* and *episodic*) for positive symptoms and four distinct trajectories (*response*, *delayed response*, *relapse*, and *no response*) for negative symptoms. Two distinct trajectories of the *response* and *delayed response trajectories* in OPUS cohort study [9] could be similarly identified as *early response and stable* and *delayed response trajectories* in our study. A study conducted by Pelayo-Teran et al [20] found five distinct trajectories for positive and negative symptoms. Case et al [13] found four different trajectories for the PANSS total score. Another study by Levine and Robinowitz [10] found five distinct trajectories for PANSS positive symptoms with one group that had a rapid and considerable improvement, and four other distinct trajectories differentiated by their severity of the scores over time. Differences in the findings between our study and three of the above studies [10, 13, 20] could be due to the fact that the latter studies had used data from clinical trials, and analysed it using different statistical methodologies [10, 13]. The studies also differed in terms of the scales used, i.e., the other studies used the Scales for Assessment of Negative (SANS) and Scales for Assessment of Positive Symptoms (SAPS) [9, 20] for measurement of symptom severity. Our study is more similar in terms of statistical methodology and profile of patients to that by Austin et al., where the patients recruited belonged to an early intervention service [9]. However comparisons should be viewed with a degree of caution because their study had an extended follow up of 10 years with a relatively smaller sample size (n = 496), a third of the sample had substance abuse diagnosis at baseline, and, they did not incorporate general psychopathology symptoms in their analysis.

Generally, more than 80% of our patients identified as *early response and stable trajectory* experienced a reduction and stabilization of severity of positive, negative and general psychopathology symptoms over the 2 years follow up. The current findings are consistent with our previous findings that higher remission rates were observed in this sample[3]. The rates of early improvement in symptoms which then stabilises in our sample are higher than that reported in other studies. This could be due to the fact that the EPIP programme has adopted a multidisciplinary approach which provides comprehensive, integrated, patient-centred care with access to a wide range of psychosocial interventions including case management, family therapy, cognitive behaviour therapy and vocational rehabilitation which may have benefited the patients [3]. Cultural factors such as family support could also be a possible explanation for these differences. In our study, majority of our patients (89.8%) lived with their family members who might play an important role in providing physical and mental health support to the patients aiding their recovery. Another possible reason is that the prevalence of substance abuse in schizophrenia patients in Singapore is lower than that found in studies conducted in the West [26]. Earlier studies have suggested that a low level of substance abuse in schizophrenia patients is associated with less frequent relapse and good treatment response. However, a sub-group of patients showed less than optimal treatment response as indicated by *early response and relapse*, *slower response and no response*, and *delayed response trajectories* in positive, negative and general psychopathology symptoms similar to the study by Rabinowitz et al who identified a deteriorating course of symptom severity in a small group of patients[27].

Our four distinct trajectories identified from negative symptoms appear to be comparable to the four trajectories found in the OPUS cohort study [9]. Majority (84.4%) of our patients belong to *early response and stable trajectory* which is characterized by low level of negative symptoms at early stage, initial decrease and stabilization over 2 years follow-up. However, although the pattern of this trajectory seems to be similar, the proportion of patients in the trajectories differed. The OPUS study observed that only 28% of their patients show a reduction of negative symptoms over the first two years followed by stabilization over the remaining eight years. They had used a different measure i.e. the SANS-SAPS scales to assess negative symptoms in their sample. This could possibly have led to some of the differences observed in the two studies.

As compared to the *early response and stable trajectory*, poor symptom trajectories including *early response and relapse trajectory*, *slower response and no response trajectory*, and *delayed response trajectory* in positive, negative and general psychopathology symptoms were predicted by younger age, male gender, unemployed and economically inactive status, lower education, longer DUP and diagnoses of schizophrenia spectrum and delusional disorders. The influence of male gender on *delayed response trajectory* was supported with the current body of research, suggesting that being male was associated with a poorer trajectory membership [18, 28–30]. This is consistent with our earlier study [28] that female patients showed slightly better improvement in symptoms, functional remission and recovery compared with male patients who attended the early psychosis intervention programme. It is possible that men are more noncompliant than women to treatment which subsequently influences their symptoms trajectory over time [5]. It is also possible that these gender differences exist due to differences in the reporting of symptoms among the two groups which may be influenced by societal expectations of behaviour in men and women in an Asian society. We found that longer DUP was associated with *delayed response trajectory* for positive and negative symptoms, *early response and relapse trajectory* for negative symptoms and general psychopathology symptoms and *slower response and no response* for negative symptoms. Previous studies have shown that longer DUP was associated with poorer response to treatment on several measures [3, 20, 31]. For example Pelayo-Teran et al found that longer DUP was associated with trajectories of worse outcome such as *non-responders*, *partial responders* and *slow partial responders trajectories* for positive symptoms. In the OPUS cohort study, longer DUP was a significant predictor for *relapse*, *delayed*, *episodic* and *no response trajectories* for positive symptoms but not for negative symptoms. Interestingly, previous studies [9, 20] which tried to determine the relationship between longer DUP and poor negative symptoms trajectories have found that the relationship was not significant. This may be due to fact that in the study by Pelayo-Teran et al [20] the subjects had a chronic illness and were enrolled at different stages of antipsychotic response, moreover the negative symptoms were assessed using a different measure (SANS-SAPS scales). In relation to functioning trajectories, a substantial proportion of the sample presented with *high functioning trajectory* (63.9%) as well as *moderately stable functioning trajectory* (33%). Only a small proportion of the sample had *deterioration in functioning trajectory* (3.1%). These findings are contradictory to Hodgekins et al. [15] who reported that a large proportion of individuals displayed a high level of social disability which did not improve over the first 12-month period (66%). However, they had used the ‘Time Use Survey’ to measure social functioning in terms of weekly hours engaged in structured activity which can directly compare functioning of clinical samples with non-clinical norms [15]. As compared to *early response and stable trajectory*, patients belonging to other trajectories either in negative, positive and general psychopathology symptoms were more likely to have *deterioration in functioning trajectory* in GAF scores over time. These findings support the suggestion that it is important to focus on symptoms trajectories since the reduction of psychopathology is linked to improved functioning and ultimately clinical recovery [32]. However, it must be kept in mind that our findings do not imply causality because the nature and direction of relationship between symptoms and functioning trajectories were not established (e.g., improvements in functioning might also improve symptoms). Limitations to the present study should be noted while interpreting the results. First, the study does not take into account medication effect on symptom trajectory and subsequently on functioning. Second, we also noted a higher non-response rate at 2 years follow-up which may have affected the generalization of the study findings. Notwithstanding these limitations, there were several strengths in the study. To the best of our knowledge, this is first Asian study assessing the latent trajectory of symptoms and functioning in a first episode psychosis population. This study was conducted at a single site of a tertiary mental health centre in Singapore, and the patients were assessed and diagnosed by trained psychiatrists and case managers. With regards to clinical implications, this study suggests that there are small groups of patients who may be at a higher risk of deteriorating symptom severity than others. These three groups comprised *early response and relapse*, *slower response and no response*, and *delayed response trajectories*. The findings also suggest that those who are younger, male, unemployed, economically inactive, with lower education, longer DUP and those diagnosed with schizophrenia spectrum and delusional disorders may be at risk of deteriorating symptoms over the period of follow-up. Knowledge about the characteristics of these trajectories as well as the risk factors and their association with poor functioning outcomes can enable clinicians and therapists to modify treatment strategies in order to enhance the likelihood of recovery. Extending treatment follow-up has also been proposed [9, 33] to gain a better understanding of these trajectories and their outcomes. For example Melchior et al, [33] have suggested that patients who present with delayed response should be followed-up over a longer period of time than patients in the other subgroups to confirm whether their symptom response reduces further or deteriorates, which we felt could also be applied to other group trajectories (*early response and relapse* and *slower response and no response*). In conclusion, our findings indicate that distinct group trajectories of patients in response to treatment can be identified using latent class growth analyses. Future studies are needed to explore treatment strategies that are tailored to the specific needs of patients who do not benefit sufficiently from standard treatment including extending the treatment follow up.
